# Reliability and validity of a Chinese version of PERMA-Profiler for left-behind children in rural China

**DOI:** 10.1186/s41155-026-00389-y

**Published:** 2026-04-11

**Authors:** Yujia Ren, Menglong Li

**Affiliations:** Physical Education Institute, Hunan First Normal University, No.1015, The Third Fenglin Road, Changsha, 410205 China

**Keywords:** PERMA-Profiler, Reliability, Validity, Left-behind children in rural China

## Abstract

**Objective:**

To examine the reliability and validity of a Chinese version of PERMA-Profiler for left-behind children in rural China, and to provide a reliable tool for measuring their well-being.

**Methods:**

Brislin’s model was used to form a Chinese version of the PERMA-Profiler through translation, backward translation and cross-cultural adaptation. A survey was then conducted on 802 left-behind children selected using convenience sampling from rural schools in Hunan Province, China. Evidence of reliability of the test scores was evaluated by its internal consistency and test–retest reliability, validity of the test scores was evaluated by its internal structure and relations to other variables.

**Results:**

The Chinese version of PERMA-Profiler included a total of 15 items in 5 dimensions. The Cronbach's *α* coefficient of the total score of the scale was .888; the Cronbach’s *α* coefficients of all dimensions ranged from .78 to .80; its test–retest reliability was .85. The results of confirmatory factor analysis (CFA) show that the fitting indexes of the model met the standard. The evidence based on relations to other variables show a significant positive correlation between the scale and the gold standard, indicating good validity of the scale.

**Conclusion:**

The Chinese version of PERMA-Profiler has good reliability and validity, and can be used for evaluating Chinese left-behind children’s well-being.

## Introduction

Well-being is the ultimate goal of human development. Philosophers began exploring well-being over two thousand years ago, shaping two predominant schools of thought: hedonism and utilitarianism. Psychologist Wilson proposed the notion of subjective well-being (SWB), which regards well-being as the sum of positive emotions, negative emotions and quality of life (Diener, 1984). Subsequently, the academic community put forward the concept of psychological well-being (PWB), which believes that well-being is the acquisition of coordinated consistency in self and meaning in life and that well-being is associated with stronger power driving internal growth (Ryff & Keyes, 1995). Afterwards, subjective well-being and psychological well-being have gradually become the mainstream topics of research on well-being, expanding the direction for research on well-being. However, they have also begun to merge due to their inability to fully explain well-being.

Professor Seligman, the father of positive psychology and president of the American Psychological Association, proposed PERMA theory (Seligman, 2018) by integrating different dimensions of well-being, constructing a well-established and multidimensional framework of well-being. PERMA theory suggests that the acquisition of well-being involves five factors: positive emotion, engagement, relationship, meaning, and accomplishment Cabrera & Donaldson, 2024; Seligman, 2018). Specific, positive emotion (P) refers to the individual’s pleasurable experiences, covering positive psychological perceptions such as pleasure, joy, warmth, and comfort. Engagement (E) refers to the individual’s devoted focus on behaviors or activities that are inherently attractive, where he loses self-awareness and temporarily does not perceive the passage of time. Relationship (R) highlights the presentation and maintenance of a positive, high-quality communication and connection with others and the social environment. Meaning (M) is defined as a kind of self-breakthrough and transcendence, and is associated with the direction of life and personal values. Accomplishment (A) represents the sense of achievement from experiences such as winning, honor, and skill acquisition (Cabrera & Donaldson, 2024; Seligman, 2018). Individuals can obtain well-being by fulfilling one or some of these factors, but will move towards prosperity when all of them are satisfied. PERMA theory emphasizes the aspiration for prosperity and the ultimate goal of life, manifested in the pursuit of a high-level, wonderful life, which is in line with China's effort to continuously enhance people's sense of achievement, well-being, and security (Zhang & Guo, 2019).

With the acceleration of social changes in China such as urban–rural development and economic transformation, a large number of workers have left their hometowns to work in cities, resulting in a large number of left-behind children in most rural areas (Li & Ren, 2024; Wan & Ren, 2023). These children face many difficulties due to the lack of parental companionship and care. Their sense of well-being has therefore become a focus of social attention (Wen et al., 2021). For example, it has been found that many factors in the social and family environments prevent left-behind children from receiving sufficient education and cause different problems in their growth. Hence, left-behind children’s well-being needs to be taken seriously by schools and all sectors of society (Li et al., 2021; Qu et al., 2021; Zhou et al., 2020). Currently in China, left-behind children’s level of well-being haven’t been evaluated by PERMA theory (Wang & Zhou, 2017; Zhao et al., 2018) and comprehensively described mainly based on subjective well-being proposed by Dinner et al. (Diener, 1984; Diener et al., 2018; Larsen et al., 1985) or psychological well-being proposed by Ryff and Keyes (1995).

PERMA-Profiler, compiled by Butler and Kern (2016) based on PERMA theory, is currently one of the most comprehensive and reliable tools for measuring personal well-being and has been proven in multiple studies to have good reliability and validity. For example, PERMA-Profiler has been widely used in empirical studies by researchers in the United States, revised in multiple cultural contexts such as Australia, Japan, and South Korea, applied to groups such as teachers, corporate employees, and the elderly, and found to have good reliability and validity and can reflect well-being from multiple dimensions (Chaves et al., 2023; Paniagua-Granados et al., 2024; Ryan et al., 2019; Wagner et al., 2020). The scale contains five dimensions: positive emotion (P), engagement (E), relationship (R), meaning (M) and accomplishment (A). It has a relatively small number of items, making it easy to operate. PERMA-Profiler was initially made in the context of Western culture, and has been validated by numerous researchers through empirical studies to have good reliability and validity, thus receiving widespread attention and application. Yang et al. (2022), Wagner et al. (2020), Umucu et al. (2021) and other scholars conducted empirical research using PERMA-Profiler, while Bartholomaeus et al. (2020), Umucu et al. (2021), Giangrasso (2021) and other researchers performed multiple rounds of reliability and validity tests on this scale. Besides, PERMA-Profiler has also been translated into multiple languages, widely utilized internationally, and found to have good adaptability among student populations. For example, Kovich et al. (2023) conducted a survey on 43,000 college students and found that PERMA-Profiler has good reliability and validity in its application among college students. However, there are currently no relevant research reports on its application among left-behind children in rural China. Hence, Butler and Kern's (2016) PERMA-Profiler was incorporated in this study to test and examine its reliability and validity among left-behind children in rural China, so as to provide a reliable tool for measuring the well-being of left-behind children in rural China.

## Methods

### Participants

From March to May 2024, 850 questionnaires were distributed offline to left-behind children selected via convenience sampling from 5 rural schools in western and central Hunan Province, China. A total of 836 questionnaires were collected, of which 802 were valid. The sample consisted of 413 boys and 389 girls, with ages ranging from 8 to 12 years (M = 10.03, SD = 1.20). Two weeks later, 50 children were selected from the initial 802 participants for a retest. The inclusion criteria for the research participants are as follows: (1) those aged below 16 years old; and (2) those with both or one of their parents leaving their hometowns and families to work in cities. Consent was obtained from the research participants and their school administrators, and all the research participants participated in this survey voluntarily. This survey was approved by the Ethics Committee of Hunan First Normal University.

The three junior middle schools that were contacted in advance declined to participate in the survey due to scheduling conflicts with their teaching and administrative arrangements. Consequently, five rural primary schools were ultimately included in the study. Considering that ages 8–12 represented a critical period affected by the experience of parental absence and was characterized by a high incidence of left-behind phenomena (Zhou & Su, 2023), this age group offered strong representativeness. Furthermore, children aged 8–12 have developed basic reading comprehension skills, allowing them to better understand the questionnaire items and complete self-assessments (Lin, 2018). Thus, the final age range for this study's sample is 8–12 years, corresponding to grades 3–6 in the primary school stage of the Chinese education system.

### Tools

1. Demographic Data Scale. This scale covers information such as gender, grade, parents' marital status, parents' educational level, parents' state of migrating for work and parents' years of migrating for work.
2. PERMA-Profiler (Butler & Kern, 2016). This scale was compiled by Butler and Kern’s (2016) to assess an individual's level of well-being. The scale covers a total of 15 items in five dimensions: positive emotion (e.g. How often do you feel positive?), engagement (e.g. How often do you become absorbed in what you are doing?), relationship (e.g. To what extent have you been feeling loved?), meaning (e.g. To what extent do you lead a purposeful and meaningful life?) and accomplishment (e.g. How often do you achieve the important goals you have set for yourself?). It adopts Likert 10-level scoring (0–9 points) for each item, with 0 indicating "completely disagree" and 9 indicating "completely agree". A higher score suggests that the individual has a stronger sense of well-being. The scores from this scale have demonstrated good evidence of reliability and validity.
3. Index of Well-Being (IWB) (Campbell, 1976). In view of the lack of a standardized tool for assessing left-behind children's well-being, IWB, which has good reliability and validity and has been widely used, was used in this study as the validity criterion. IWB was compiled by Campbell (1976) and consists of two parts: overall sentiment index (8 items) and overall life satisfaction (1 item). They are weighted to obtain the overall well-being index. A higher score indicates a stronger sense of well-being. The Cronbach's α coefficient of this scale is.93 (Huang & Xing, 2005). The scores from this scale have demonstrated good evidence of reliability and validity.

### Procedure

#### Translation and backward translation

After contacting the original author via e-mails and obtaining authorization, PERMA-Profiler was accurately translated into Chinese following the five-stage procedure proposed by Brislin (1970), where cross-cultural adaptability and academic rigor were ensured. This procedure was performed according to the practice of predecessors (Wang & Xia, 2016): (1) Preliminary Translation. This stage involved independent translation by two experts: a postgraduate proficient in both languages with a doctor's degree in psychology and a professional translator with the background in English and the certificate in Test for English Majors-Band 8. They translated the original English version (E0) separately and produced two independent Chinese versions (C1 and C2). (2) Synthesis and Review. A medical doctor with overseas learning experience was invited to carefully compare and revise the two Chinese versions (C1 and C2), aiming to integrate and form an optimized Chinese version (C3) through unifying terminology, eliminating ambiguity, and ensuring that the Chinese version truly and fully reflects the meaning of the original version. (3) Backward Translation (BT). Two other researchers with high-level bilingual abilities intervened and respectively translated the Chinese version (C3) back into E1 and E2 in English, for the purpose of examining the accuracy of translation from a reverse perspective. (4) Consolidation. E1 and E2 were compared by two backward translators; with the assistance of a third associate professor of psychology with bilingual abilities, they discussed the differences in depth and made revisions to jointly identify the final backward translated version (E3). (5) Comparative Analysis and Finalization: All the researchers involved in translation and backward translation collectively reviewed all translated versions, conducted detailed discussions on each issue, and made sure that the final Chinese version was highly consistent with the original scale in English in terms of content, context, and intention, so as to ensure precise localization and cultural adaptability of the scale.

#### Cross-cultural adaptation

(1) Expert Consultation. 6 senior professional researchers were specially invited to form a review team in charge of ensuring the scientificity and applicability of the Chinese version of the scale from multiple perspectives. The team comprised two PhDs in medical statistics, two professors of medical psychology, two professors of education, and several frontline teachers and school administrators who work with left-behind children. The team conducted an in-depth comparison and analysis of the original English version (E0), preliminary Chinese version (C3) and backward translated English version (E3). Their review focused on fitting in each dimension, the accuracy of terminological expressions, and whether the wordings were in line with the local cultural context of China. They proposed constructive revision suggestions under the premise of maintaining the original meanings. According to expert consultation and recommendations, Item 9 "You are satisfied with your interpersonal relationships" was revised into "You are satisfied with your relationships with family members, friends, classmates, and teachers". (2) Pre-survey Implementation and Scale Optimization. In order to verify the practical applicability of the revised scale, the inclusion and exclusion criteria were strictly followed in this study using convenience sampling to conduct a pre-survey 4 weeks before the formal survey. According to the statistical principle that the sample size should be 5–10 times the number of items in the scale (Wu, 2010), and taking into account the needs of questionnaire analysis, 120 left-behind children who met the inclusion criteria were screened in a rural primary school in Hunan Province for pre-survey, and 112 valid questionnaires (93.3%) were collected. During the pre-survey process, the participants’ understanding and feedback on the scale's instructions, the content of each item, and response manners were meticulously recorded. When most children use the 0–10 point scoring method, it is difficult to accurately distinguish the differences between 6 and 7 points, 8 points and 9 points, and they are prone to obvious hesitation and confusion in the process of answering. In view of the fact that the abstract thinking and fine discrimination ability of children in primary school are not yet fully mature (Lin, 2018), in order to reduce the cognitive load caused by too many levels and ensure the authenticity and effectiveness of the evaluation data, Likert 10-level scoring used in the original scale was changed to Likert 5-level scoring (1–5 points) for each item, with 1 indicating “completely disagree” and 5 indicating "completely agree". A higher score indicates a stronger sense of well-being in the left-behind child. Thus, the Chinese scale (C4) was formed after further enhancing the accuracy and cultural adaptability of the scale content. An in-depth analysis was performed on the pre-survey data to identify and address potential understanding barriers, and targeted adjustments were made to wordings in the Chinese version of the scale (C4) to enhance its readability and comprehensibility. Finally, the Chinese version of PERMA-Profiler maintaining the original 15 item structure.

#### Data collection methods

Eight rural primary and middle schools with a high proportion of left-behind children and a willing to cooperate in the research were selected as survey sites. Among them, three junior middle schools were unable to conduct the survey for certain reasons, and the final questionnaires were administered in five rural primary schools. Prior to the survey, consent was obtained from the schools, guardians and the students themselves. During administration, the principle of voluntary participation was upheld, allowing students to opt out at any stage. Only those who had received approval and voluntarily participated were included in the study. The assessments were conducted in classrooms or conference rooms at each school. For data collection, paper-based questionnaires were used for collective responses. The research team provided brief training, explaining the instructions and precautions for filling out the questionnaires. The survey was conducted anonymously, with questionnaires filled out and collected on-site and completeness checked one by one, taking about 8–12 min to complete. To assess the test–retest reliability of the scale, 50 participants were randomly selected from the total sample for a second test four weeks after the initial assessment. The retest sample was selected using a random number table method, and the retest questionnaires were completed in the same environment within the same schools, following the same procedures as the initial assessment.

### Statistical methods

SPSS 22.0 software was used for data entry. The data were checked by two researchers two rounds to ensure their accuracy and correctness. SPSS and AMOS software were used for analysis to cover multiple key aspects of scale-based evaluation. (1) Use of Cronbach's *α* Coefficient for Internal Consistency Reliability Evaluation. Fifty individuals were randomly selected from the 802 participants to complete the questionnaire a second time after two weeks. The correlation between the results obtained these two times was calculated. The intraclass correlation coefficient (ICC) >.70 indicates good stability and good test–retest reliability of the scale (Terwee et al., 2007). (2) Confirmatory Factor Analysis. Confirmatory factor analysis (CFA) with maximum likelihood estimation was conducted to test whether the pre-specified theoretical model fit the data, thereby evaluate the internal structure of the scale.

## Results

### Item analysis

The purpose of item analysis is to determine whether items included in the scale are valid and appropriate. The principle is to first sum up analysis items, divide them into the high-score and low-score groups (with 27% and 73% quantiles as the boundaries), and then use *t*-test to compare the high-score and low-score groups. If there is a difference, it indicates that the item is appropriate; conversely, it indicates that the item cannot distinguish information, is unreasonable, and should be deleted (Zhang & Zhou, 2020). In this study, a total of 15 items (p1-p15) were analyzed. After summing up these 15 items, they were divided into the high-score and low-score groups. *t*-test was used to examine their differences. From Table 1, it can be seen that both the high-score and low-score groups had significant differences (*P* <.05) for all the 15 items, indicating that all the 15 items have good discriminability and should be retained. Therefore, the analysis items should not be deleted.

**Table 1:** Results of item analysis (Discriminability)

	Group (mean ± standard deviation)		*t (Decision value)*	*p*
	Low-score group (*n* = 230)	High-score group (*n* = 220)		
p1	2.97 ± 0.22	4.74 ± 0.47	20.47	<.001
p2	3.05 ± 0.23	4.72 ± 0.45	19.26	<.001
p3	2.99 ± 0.27	4.75 ± 0.43	20.00	<.001
p4	2.69 ± 0.33	4.68 ± 0.48	22.90	<.001
p5	2.87 ± 0.25	4.74 ± 0.46	21.10	<.001
p6	2.80 ± 0.24	4.70 ± 0.47	21.57	<.001
p7	2.79 ± 0.39	4.63 ± 0.49	21.55	<.001
p8	2.68 ± 0.29	4.70 ± 0.50	22.11	<.001
p9	2.61 ± 0.25	4.81 ± 0.41	25.27	<.001
p10	3.11 ± 0.26	4.72 ± 0.45	18.28	<.001
p11	3.04 ± 0.38	4.75 ± 0.43	20.52	<.001
p12	3.12 ± 0.27	4.81 ± 0.39	19.25	<.001
p13	3.03 ± 0.36	4.74 ± 0.44	19.36	<.001
p14	3.00 ± 0.34	4.70 ± 0.46	19.42	<.001
p15	2.98 ± 0.34	4.76 ± 0.43	22.11	<.001

### Validity analysis

#### Confirmatory factor analysis

Based on the five-factor structure of the PERMA-Profiler, CFA was used to examine the internal structure of the measurement model. The model fitting parameters obtained by applying the maximum likelihood method are shown in Table 2. The value of χ^2^/df was 2.733 < 3; NFI, RFI, IFI, TLI, CFI, and GFI were all greater than.90; RMSEA was.057 <.080. The results of confirmatory factor analysis indicate high goodness of fit of the model (Hou et al., 2004).

**Table 2:** Model fitting results from confirmatory factor analysis

Model fitting	**χ**^**2**^**/df**	**NFI**	**RFI**	**IFI**	**TLI**	**CFI**	**GFI**	**RMSEA**
Fitting results	2.733	.902	.911	.925	.911	.921	.909	.057
Reference standard	< 3	>.90	>.90	>.90	>.90	>.90	>.90	<.08

#### Relations to other variables

The relationships to other variables of the scale were examined by analyzing the correlation between the total score of the PERMA-Profiler and that of the criterion measure. The Pearson correlation coefficient was used to indicate the strength of the association. As recommended by Andresen (2000), the correlation coefficients were rated as follows: excellent (≥.60), adequate (.30-.59), and poor (≤.29). The analysis results show that the correlation coefficient between the total score of PERMA-Profiler and criterion scale is.61, indicating good validity of the scale revised in this study for measuring left-behind children's well-being.

### Scale reliability

Cronbach's *α* test was performed to assess the reliability of the scale; corrected item-total correlation (CITC) was used to measure the reliability of each item. The results show that the Cronbach's *α* coefficient of this scale was.89; the Cronbach's *α* coefficients corresponding to the five factors were.78,.80,.80,.79 and.78, respectively. The Cronbach's *α* coefficient of each variable was greater than.70 in all cases. In addition, the CITC values of each item and the Cronbach's *α* coefficients of the deleted items met the research requirements, indicating high stability of each variable in the scale and good internal consistency reliability (Table 3). The results of data analysis of the two measurements show that the ICC was.85, indicating good stability and good test–retest reliability of the scale (Terwee et al., 2007).

**Table 3:** Analysis of internal consistency reliability

Factor	Item	Corrected item-total correlation	Cronbach's *α* after item deletion	Cronbach's *α*
F1	p1	.61	.70	.78
	p2	.60	.72	
	p3	.64	.68	
F2	p4	.66	.71	.80
	p5	.62	.75	
	p6	.65	.72	
F3	p7	.64	.74	.80
	p8	.65	.73	
	p9	.66	.73	
F4	p10	.62	.74	.79
	p11	.64	.72	
	p12	.66	.70	
F5	p13	.61	.72	.78
	p14	.62	.71	
	p15	.64	.68	

## Discussion

### High application value of the Chinese version of PERMA-Profiler

The revised Chinese version of PERMA-Profiler was created through rigorous translation, backward translation, cultural adaptation and pre-investigation. It includes 15 items in 5 dimensions: positive emotion, engagement, relationship, meaning and accomplishment. The scale is more specific in content to issues related to left-behind children’s well-being. Compared with existing universal well-being scales, our scale aligned closely with the life circumstances of left-behind children, particularly focusing on peer and grandparent support in the "relationships" dimension in the context of parent–child separation, while emphasizing the understanding and acceptance of the left-behind life in the "meaning" dimension. Therefore, this scale can provide a scientifically reliable and easy-to-use assessment tool for measuring left-behind children’s well-being and related studies.

The Chinese version of PERMA-Profiler has high application value, mainly reflected in the following aspects: First, it is a highly targeted scale. Specifically designed for the specific group of left-behind children, the scale takes into account their unique needs and challenges in terms of positive emotion, engagement, relationship, meaning and accomplishment, and is therefore useful to accurately assess their well-being state. This study introduces the first PERMA tool specifically tailored for rural left-behind children in China, laying a foundation for subsequent research. Secondly, it has multidimensional coverage. Through the five dimensions including positive emotion, engagement, relationship, meaning and accomplishment, it can comprehensively reflect the complexity of left-behind children's well-being, avoid one-sided evaluation (for instance, the "relationships" dimension can assess whether peer support compensated for the absence of parents, while the "achievement" dimension can reflect how academic performance affected self-worth), and facilitate a deeper understanding of the multiple factors that affect well-being. Thirdly, it is easy to operate and use. The concise and clear design of the scale (with a total of 15 questions, and the scoring method is adjusted to 5 grades, which reduces the cognitive burden of rural children) makes it easy to implement in rural areas or schools with limited resources. It can be easily used by educators, social workers and researchers at low implementation difficulty and costs, resulting in higher application universality and practicality of this scale. Fourth, it promotes policy formulation and intervention. Data obtained using the scale can provide an empirical basis for governments, research institutions and social organizations, help decision-makers identify key factors affecting left-behind children's well-being (For example, a low score of "relationship" may indicate insufficient social support), and enable them to develop more accurate and effective policy measures and intervention schemes, such as strengthen the psychological guidance of boarding students, etc. Fifth, it is useful for evaluating intervention effectiveness. The scale can be used for pre- and post-test comparison to evaluate the actual effects of different intervention measures on left-behind children's well-being, provide quantitative basis for continuous improvement and optimization of related services, and ensuring the effective utilization of resources. In summary, the Chinese version of PERMA-Profiler has important theoretical and practical application value, since it not only provides a scientific basis for assessing left-behind children's well-being, but also show its unique value in 3 aspects: theoretical fit, group pertinence and practical feasibility.

### Scientificity of the Chinese version of PERMA-Profiler

Cronbach's α coefficient was used in this study to evaluate the internal consistency reliability of the scale, which served as the standard for measuring the reliability of the scale. When the Cronbach's *α* coefficient exceeds.80, it indicates that the scale has excellent reliability (Zhang & Zhou, 2020). The overall Cronbach's *α* coefficient of the Chinese version of PERMA-Profiler for measuring left-behind children's well-being reached.85, with coefficients in all dimensions ranging from.78 to.80. Although the coefficients of some subscales were slightly lower than the ideal level of.80, the overall scale performed well, indicating good internal consistency of the scale. In such a highly heterogeneous group as left-behind children, considering the diverse age ranges and types of left-behind, the scale still achieved this level of reliability, indicating good homogeneity among the items and reliable measurement of various aspects of well-being. The test–retest reliability reached.85, further indicating that the scale scores remained stable over the 4-week interval, suggesting that the measurement captured a relatively lasting quality of well-being, rather than transient emotional fluctuations.

The validity of the scale was evaluated by internal structure and relations to other variables. Confirmatory factor analysis was used to test the 5-factor structure of the scale, the results show that the χ^2^/df value was 2.733, below the standard of 3. Besides, a number of fitting indexes such as NFI, RFI, IFI, TLI, CFI and GFI all exceeded.90, and RMSEA was.057, below.080, indicating good fitting and good internal structure of the model (Ren et al., 2021). This result not only verifies the applicability of PERMA theory in the group of left-behind children in China, but also shows that the 5-factor structure of happiness has cross-cultural stability. Relations to other variables serve as an important indicator for evaluating the validity of a scale. In this study, IWB was selected as the criterion. Correlation analysis revealed a significant positive correlation between the Chinese version of the PERMA-Profiler and IWB, with a correlation coefficient exceeding.60. This indicates that the Chinese version of the PERMA-Profiler demonstrates high consistency with IWB when assessing the well-being of rural left-behind children in China, making it an effective tool for evaluating their well-being (Chen et al., 2020). This finding enriches the theoretical framework of well-being and encourages the measurement of well-being to develop in more comprehensive dimensions.

In summary, the Chinese version of PERMA-Profiler was found to perform well in terms of reliability and validity. It not only has high internal consistency and good stability, but also meets the standards for internal structure and relations to other variables, providing a reliable tool for subsequent empirical research and policy formulation.

### Limitations and directions for future research

There are still several limitations in this study. Firstly, the sample selected for this study was limited to Hunan Province, China, and convenience sampling may limit the diversity of the sample obtained and promotion of research conclusions to other regions. Secondly, the retest sample consisted of only 50 individuals, which may somewhat affect the evaluation of the test–retest reliability. Thirdly, the research centered on left-behind children in the primary school stage (ages 8–12). Although this age group constituted a larger proportion of the left-behind population and was a critical period for psychological development, it did not cover the adolescent group aged 13–15, limiting the applicability of the scale across a broader age range.

Given the above limitations, future research should focus on three points: firstly, optimizing the sampling method to overcome the current limitation of selecting samples from Hunan only, by including left-behind children from different regions in eastern, central and Western China, employing a stratified random sampling method to enhance the representativeness of samples. Secondly, expanding the size of the retest sample and appropriately extending the interval between retests to more accurately evaluate the test–retest reliability of the scale. Thirdly, broadening the age range of the subjects to include left-behind children aged 13–15 and analyzing the adaptability of the scale across different age groups.

## Conclusion

This study examined the adaptability of the Chinese version of PERMA-Profiler among left-behind children in rural China. The results showed that the Chinese version of PERMA-Profiler showed good reliability and validity in evaluating the well-being of left-behind children, which laid a solid foundation for further exploring the psychological well-being of left-behind children and promoting related research.

## Data Availability

Please contact author for data requests.
